# Supplementary material for: Artificial Intelligence-Powered Whole-Slide Image Analyzer Reveals a Distinctive Distribution of Tumor-Infiltrating Lymphocytes in Neuroendocrine Neoplasms
Source: Diagnostics (Basel). 2022 Sep 27;12(10):2340. doi: 10.3390/diagnostics12102340 (PMC9600129; doi:10.3390/diagnostics12102340)

Supplementary Table S1. Number of annotated cells in the training and validation sets.

	Lymphocyte	Tumor cell
Training	465,778	1,644,697
Validation	150,041	567,781

Supplementary Table S2. Area of annotated tissues in the training and validation sets.

	Area (mm^2)		
	Tumor Area	Tumor Stroma	Background
Training	7,861	5,693	18,505
Validation	600	421	983

Supplementary Figure S1. Intratumoral tumor-infiltrating lymphocytes (TIL) density in high-grade NENs and low/intermediate-grade NENs according to the primary origin. (A) Colorectum (B) Small intestine (C) Hepatopancreatobiliary (D) Stomach (E) Lung (F) Other organs. Values in graph indicates *P* values.

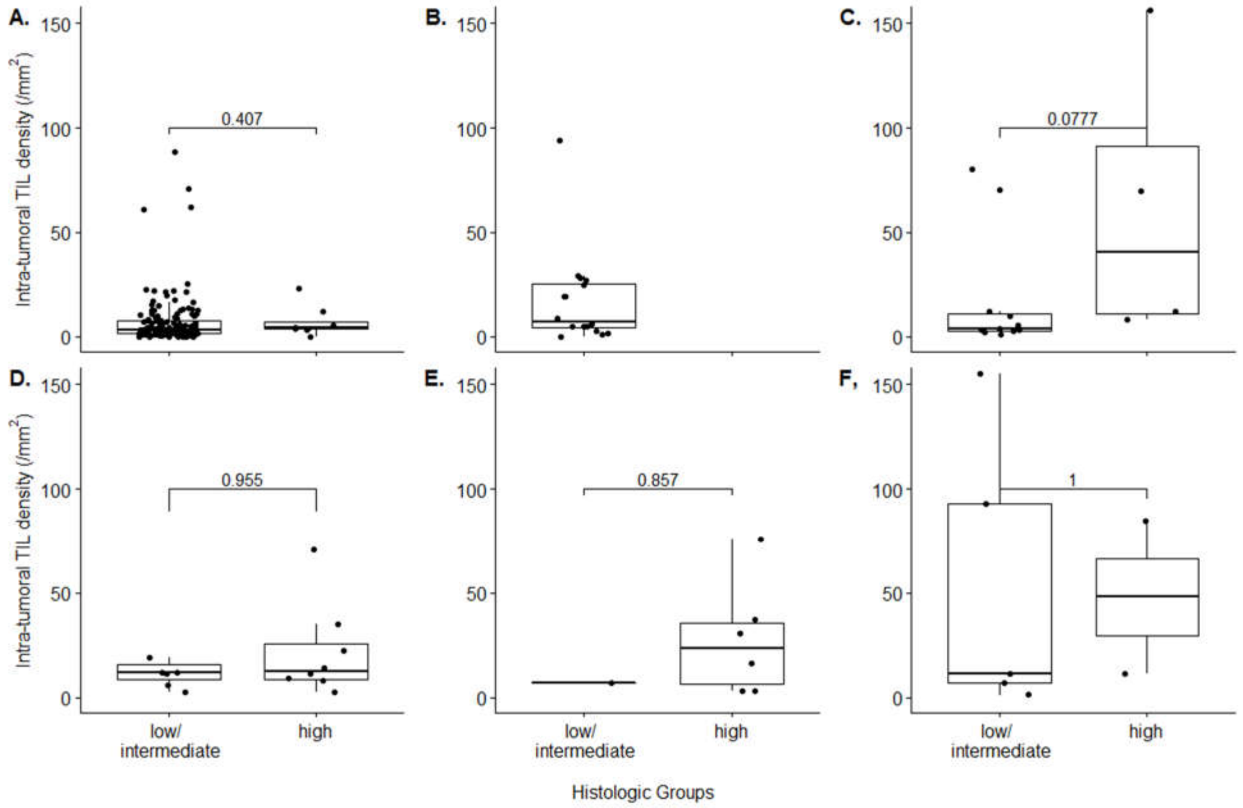

Supplementary Figure S2. PD-L1 combined positive score (CPS) in high-grade NENs and low/intermediate-grade NENs according to the primary origin. (A) Colorectum (B) Small intestine (C) Hepatopancreatobiliary (D) Stomach (E) Lung (F) Other organs.

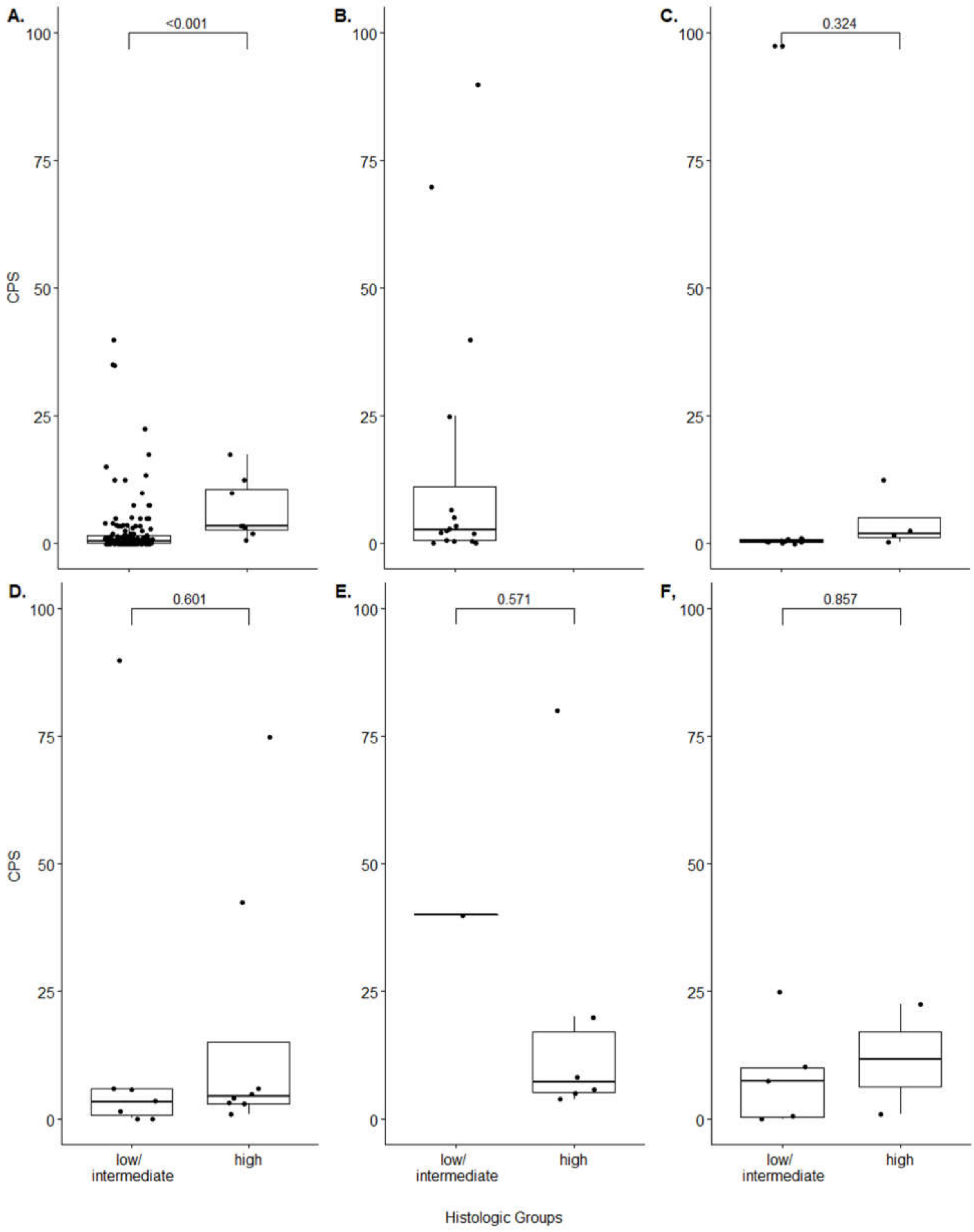

Supplement: Supplementary file 1 [file diagnostics-12-02340-s001.zip › diagnostics-1917100-supplementary.pdf]
